# Inequalities in colorectal cancer diagnosis by ethnic group: a population-level study in the English National Health Service

**DOI:** 10.1136/bmjgast-2024-001629

**Published:** 2025-01-08

**Authors:** Rebecca J Birch, Nick E Burr, John C Taylor, Amy Downing, Phil Quirke, Eva J A Morris, James Turvill, Mo Thoufeeq

**Affiliations:** 1 Pathology and Data Analytics, Leeds Institute of Medical Research, University of Leeds, Leeds, UK; 2 Leeds Institute for Data Analytics, University of Leeds, Leeds, UK; 3 Mid Yorkshire Teaching NHS Trust, Wakefield, UK; 4 Nuffield Department of Population Health, Oxford University, Oxford, UK; 5 Gastroenterology, York and Scarborough Teaching Hospitals NHS Foundation Trust, York, UK; 6 Department of Gastroenterology, Sheffield Teaching Hospitals NHS Foundation Trust, Sheffield, UK

**Keywords:** COLORECTAL CANCER, CANCER EPIDEMIOLOGY, SCREENING, PRIMARY CARE

## Abstract

**Objective:**

Studies in the USA examining the relationship between ethnicity and colorectal cancer (CRC) identified significant variation. This study sought to examine the relationship between ethnic group, route to diagnosis, early-onset CRC and stage at diagnosis in the English National Health Service.

**Methods:**

Data from COloRECTal cancer data Repository for all individuals diagnosed with CRC (International Classification of Diseases version 10, C18–C20) between 2012 and 2017. A descriptive analysis of the characteristics of the study population was performed. Multivariable logistic regression models were used to assess the association between ethnicity, route to diagnosis, stage at diagnosis and early-onset CRC.

**Results:**

Early-onset CRC was least common in those in the white ethnic group (5.5% diagnosed <50, vs 17.9% in the Asian, 15.5% in the black and 21.8% in the mixed and multiple ethnic groups, p<0.01). Diagnosis following a 2-week wait referral was significantly less common among individuals from the Asian, black, other and unknown ethnic groups than the white ethnic group (Asian OR 0.84, 95% CI 0.79 to 0.91, black OR 0.86, 95% CI 0.79 to 0.93, other OR 0.81, 95% CI 0.73 to 0.90 and unknown OR 0.70, 95% CI 0.66 to 0.73). The Asian ethnic group had significantly lower odds of emergency diagnosis than the white ethnic group (OR 0.90, 95% CI 0.83 to 0.97). Following adjustment, individuals from the Asian ethnic group were significantly less likely, than their white counterparts, to be diagnosed at stage IV (OR 0.82, 95% CI 0.76 to 0.88).

**Conclusion:**

This study identified different demographic profiles of those diagnosed with CRC between broad ethnic groups, highlighting the need to consider access to diagnostic CRC services in the context of ethnicity.

**WHAT IS ALREADY KNOWN ON THIS TOPIC**

- Data from the USA have shown higher rates of early age of onset colorectal cancer (CRC) (diagnosed before the age of 50) in individuals from non-white ethnic groups and an association between ethnicity and stage at diagnosis.

**WHAT THIS STUDY ADDS**

- It demonstrated a higher level of early-onset CRC in individuals from non-white ethnic groups in England.
- Route to diagnosis varied by ethnic group, with a higher proportion of individuals from non-white ethnic groups being diagnosed via routes other than the expedited 2-week wait route, identifying a key area for possible improvements in outcomes.

**HOW THIS STUDY MIGHT AFFECT RESEARCH, PRACTICE OR POLICY**

- Individuals from non-white ethnic groups have an earlier age of onset and higher proportions of characteristics associated with poor outcomes demonstrating the need to ensure that any interventions seeking to improve outcomes in these populations address these demographic differences.
- Ensuring access of non-white ethnic groups to the urgent suspected CRC pathway may require ethnic-specific approaches to support people and primary care in the interpretation and response to bowel symptoms.

## Introduction

 According to figures from the Office for National Statistics, 81.7% of the population of England and Wales are white, with 18.3% (8.9 million individuals) coming from non-white ethnic groups.^1^ Many studies have sought to investigate the relationship between ethnicity and cancer incidence. For colorectal cancer (CRC), incidence rates in the UK have been shown to be lower among non-white individuals than white individuals,^2^ while studies in the USA have shown the reverse to be true.^3 4^

Historical data have demonstrated that the characteristics of CRCs vary between ethnic groups. For example, tumour characteristics, such as location and stage, and patient characteristics, such as age at onset, have been shown to differ by ethnicity with individuals from non-white backgrounds being shown by some to have higher incidence of late-stage tumours and early-onset tumours.^5^,^8^ Early-onset CRC is commonly referred to as cancer diagnosed before the age of 50,^9^,^11^ studies from the USA have shown higher rates of early age of onset CRC in individuals from non-white ethnic groups.^7 8^ Studies into early-onset CRC in the UK to date have either not investigated the association with ethnicity^12^ or have focused on the relationship between ethnic group, age at onset and stage at diagnosis.^13^

Route to diagnosis is associated with cancer outcomes, with emergency diagnosis being linked to significantly worse outcomes than other routes such as screening.^14^ There is a concern that people from certain groups may have a lower participation in the bowel cancer screening programme; regional data showed women from the Asian ethnic group aged 50–69 were found to be less likely to participate in round 1 of the National Health Service (NHS) bowel screening pilot in 2000–2002.^15^ The first round of the Bowel Cancer Screening Programme in 2008 showed a lower uptake in some areas (40%) compared with an overall 52% in England.^16^ A greater ethnic mix and higher use of private healthcare were suggested as possible explanations, although this was never proven. Participation in flexible sigmoidoscopy screening was also found to be low in the Asian ethnic group.^17^ At a national level, variation in route to a cancer diagnosis has been demonstrated in relation to ethnicity across England,^18^ however, this study was restricted to individuals aged 40 and over so did not fully capture the individuals with early-onset CRC as part of this analysis.

The purpose of this study is to examine the relationship between ethnic groups and the characteristics of those diagnosed with CRC, particularly early-onset CRC, and route to diagnosis in the English NHS. Such information is important for adapting CRC screening programmes and diagnostic pathways, as it may highlight areas where additional resource and education is needed to reduce inequality. If ethnic inequalities are identified then it is vital to describe them in detail in order to tackle them and optimise diagnostic pathways, especially in non-white populations.

## Methods

### Study population and data

Data for all individuals diagnosed with CRC (International Classification of Diseases version 10 codes, C18–C20) between 1 January 2012 and 31 December 2017 were obtained from the COloRECTal cancer data Repository.^19^ The information used in this project was extracted from the National Cancer Registration and Analysis Service^20^ component of the resource. Information obtained included age at diagnosis, stage at diagnosis, tumour site (right colon (C180–C184), left colon (C185–C187), colon unspecified (C188–C189) or rectum (C19 and C20)), socioeconomic status (using the income domain of the Index of Multiple Deprivation 2015 (IMD) score), Charlson Comorbidity Score and ethnic group.

Ethnic group was obtained from the cancer registration data, which defines ethnicity using the National Disease Registration Service methodology.^21 22^ High-level ethnic groupings were used, with individuals assigned to Asian, black, white, other, mixed and multiple or unknown ethnic groups in line with the approach of the Office for National Statistics.^23^ Other studies investigating the relationship between ethnicity and cancer incidence using the same datasets have demonstrated that assigning the individuals with unknown ethnicity to one of the broad ethnic groups using the same proportions as seen in those with a known ethnicity did not significantly alter the results.^2^ Individuals with an ‘unknown’ ethnic group were included as a separate group.

#### Age at diagnosis

Age was categorised in line with the age range used for screening during the study period (early onset (<50), prescreening (50–59), screening (60–74) and postscreening (≥75)). Median age at diagnosis was calculated for each ethnic group and compared, using a nonparametric k-sample test on the equality of medians.

#### Route to diagnosis

Individuals were assigned to one of six routes to diagnosis: emergency, general practitioner (GP) referral, other hospital (a combination of inpatient and outpatient diagnoses), screening, 2-week wait (TWW) or unknown (which includes death certificate only, other and unknown routes) using the route to diagnosis fields in the cancer registration data.^24^

### Statistical analysis

χ^2^ tests were used to test for significant differences in characteristics between ethnic groups. Multivariable logistic regression models were used to assess the relationship between ethnicity, and age at diagnosis, stage at diagnosis and route to diagnosis. Adjusted ORs and 95% CIs were calculated for each variable. For age at diagnosis, a binary outcome variable was created for diagnosis before the age of 50 years and models were adjusted for ethnic group, sex, socioeconomic status and year of cancer diagnosis. Four outcome variables were created for analysis of stage at diagnosis: early stage (I or II), locally advanced (III), late stage (IV) and unknown stage. Each was modelled separately and was adjusted for ethnic group, age at diagnosis, sex, socioeconomic status, tumour site and year of diagnosis. A binary outcome variable was produced for each route (emergency, TWW, screening, GP referral, other hospital and unknown). Each route was modelled separately, with adjustment for age at diagnosis, sex, socioeconomic status, tumour site and year of diagnosis (single year). The model for diagnosis through screening was restricted to include only individuals aged 60–74, who were eligible for screening during the study period. Statistical analysis was undertaken using Stata V.16.0. The research is reported in line with the Strengthening the Reporting of Observational Studies in Epidemiology guidelines (online supplemental material).

### Patient and public involvement

The Bowel Cancer Intelligence UK Patient and Public Group was consulted and approved the study design. The results of the work have been presented at a Yorkshire Cancer Research Bowel Cancer Improvement Programme meeting, which was attended by patient representatives.

## Results

### Characteristics

Of the 202 580 individuals diagnosed with CRC in England between 2012 and 2017, 90.9% (n=184 160) belonged to the white ethnic group. Of the remaining individuals, 2.0% (n=4099) were in the Asian ethnic group, 1.5% (n=2830) were in the black ethnic group, 0.3% (n=625) were classified as belonging to mixed and multiple ethnic groups, 1.1% (n=1945) were in other ethnic groups and 4.4% (n=8921) belonged to the unknown ethnic group (table 1). Over half of the individuals in the Asian and black ethnic groups resided in deprived areas at the time of their cancer diagnosis (55.4% and 69.4% in IMD quintiles 4 and 5, respectively), compared with 34.1% of individuals in the white ethnic group (p<0.01) (table 1).

**Table 1:** Characteristics of the study population, by ethnic group

		White (%)	Asian (%)	Black (%)	Mixed/multiple (%)	Other (%)	Unknown (%)	P value
Age group	<50	10 050 (5.5)	734 (17.9)	438 (15.5)	136 (21.8)	336 (17.3)	664 (7.2)	<0.01
	50–59	19 890 (10.8)	774 (18.9)	599 (21.2)	124 (19.8)	339 (14.4)	1143 (12.8)	
	60–74	71 561 (38.9)	1501 (36.6)	853 (30.1)	198 (31.7)	751 (38.6)	3383 (37.9)	
	≥75	82 659 (44.9)	1090 (26.6)	940 (33.2)	167 (26.7)	529 (27.2)	3751 (42.0)	
Sex	Male	102 628 (55.7)	2315 (56.5)	1523 (53.8)	320 (51.2)	1089 (56.0)	4662 (52.3)	<0.01
	Female	81 532 (44.3)	1784 (43.5)	1307 (46.2)	305 (48.8)	856 (44.0)	4259 (47.7)	
Socioeconomic status	1–most affluent	40 299 (21.9)	476 (11.6)	143 (5.1)	94 (15.0)	292 (15.0)	2376 (26.6)	<0.01
	2	42 420 (23.0)	544 (13.3)	208 (7.3)	118 (18.9)	335 (17.2)	2158 (24.2)	
	3	38 717 (21.0)	808 (19.7)	516 (18.2)	116 (18.6)	406 (20.9)	1806 (20.2)	
	4	33 173 (18.0)	1071 (26.1)	881 (31.1)	149 (23.8)	489 (25.1)	1450 (16.3)	
	5–most deprived	29 551 (16.0)	1200 (29.3)	1082 (38.2)	148 (23.7)	423 (21.7)	1131 (12.7)	
Tumour site	Right colon	65 082 (35.3)	1234 (30.1)	1130 (39.9)	221 (35.4)	607 (31.2)	2875 (32.2)	<0.01
	Left colon	49 446 (26.8)	1111 (27.1)	783 (27.7)	169 (27.0)	555 (28.5)	2316 (26.0)	
	Colon, unspecified	7616 (4.1)	146 (3.6)	158 (5.6)	25 (4.0)	78 (4.0)	868 (9.7)	
	Rectum and rectosigmoid	62 016 (33.7)	1608 (39.2)	759 (26.8)	210 (33.6)	705 (36.2)	2862 (32.1)	
Stage at diagnosis	I	28 939 (15.7)	695 (17.0)	384 (13.6)	88 (14.1)	286 (14.7)	1266 (14.2)	<0.01
	II	44 496 (24.2)	896 (21.9)	604 (21.3)	140 (22.4)	452 (23.2)	1713 (19.2)	
	III	50 255 (27.3)	1318 (32.2)	805 (28.4)	171 (27.4)	537 (27.6)	1725 (19.3)	
	IV	42 096 (22.9)	845 (20.6)	806 (28.5)	180 (28.8)	502 (25.8)	2034 (22.8)	
	Unknown	18 375 (10.0)	348 (8.5)	231 (8.2)	46 (7.4)	168 (8.6)	2183 (24.5)	
Total		184 160	4099	2830	625	1945	8921	

Rectal tumours were most common among individuals from the Asian ethnic group (39.2 %) and least common among individuals from the black ethnic group (26.8%). In contrast, tumours of the right colon were most common among individuals in the black ethnic group (39.9%) and least common among individuals from the Asian ethnic group (30.1%) (p<0.01) (table 1).

### Age at diagnosis

**Figure 1:**
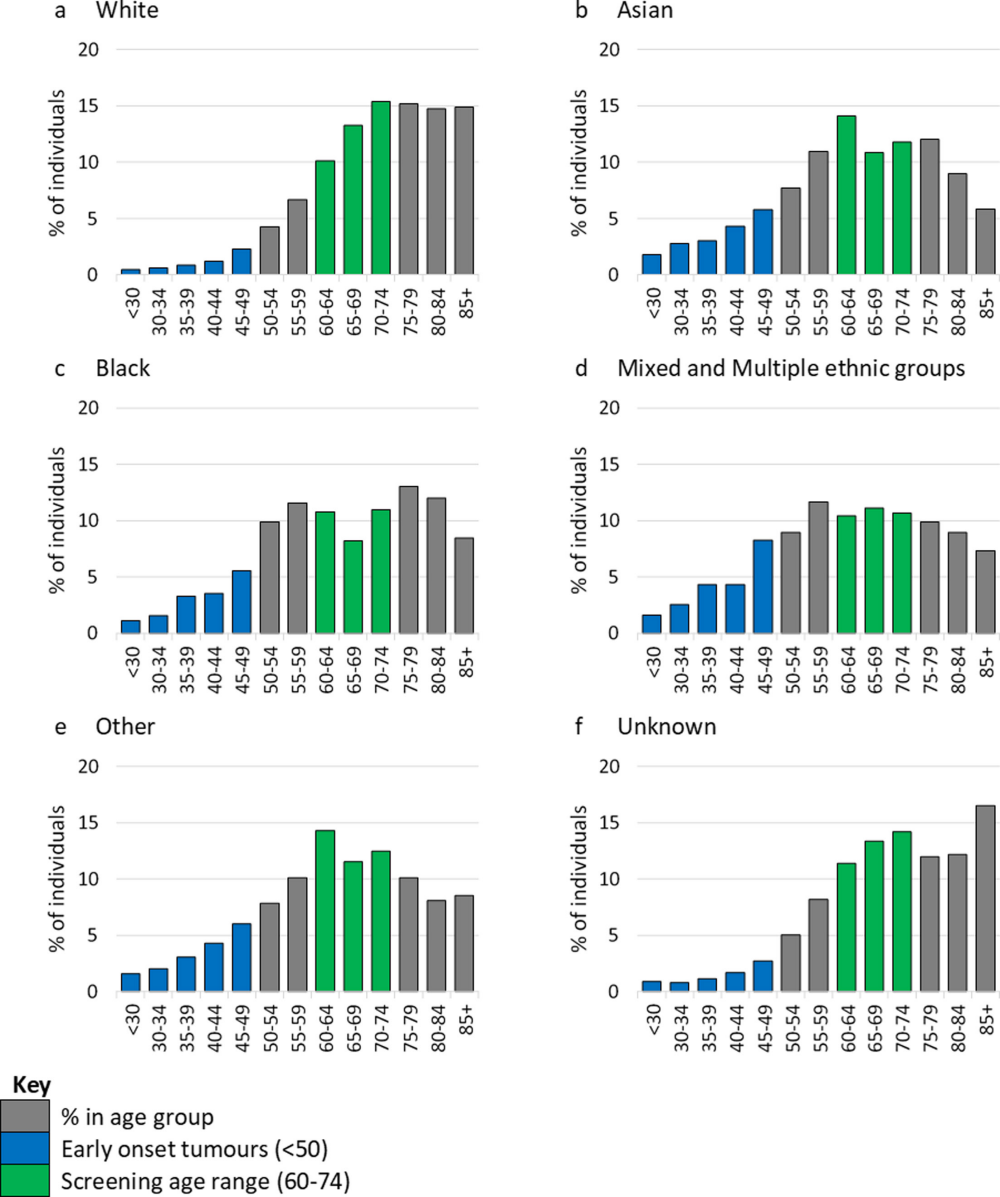
Age distribution of individuals, by ethnic group.

Early-onset CRC was more common among the non-white ethnic groups, with 17.9% of individuals from the Asian ethnic group diagnosed under the age of 50, along with 15.5% of the black ethnic group and 21.8% of those with mixed and multiple ethnic groups, compared with 5.5% of those in the white ethnic group (figure 1, table 1). Following adjustment for patient characteristics, this effect remained, with individuals from the non-white ethnic groups being significantly more likely than those from the white ethnic group to be aged under 50 at the time of their CRC diagnosis (Asian OR 3.62, 95% CI 3.33 to 3.93, black OR 2.93, 95% CI 2.64 to 3.25, mixed and multiple OR 4.66, 95% CI 3.85 to 5.64) (table 2).

**Table 2:** Results of logistic regression models for early-onset CRC (aged <50 at the time of diagnosis)

		Multivariable				Univariable			
		OR	P value	95% CI		OR	P value	95% CI	
Ethnic group	White	1.00				1.00			
	Asian	3.62	<0.01	3.33	3.93	3.78	<0.01	3.48	4.10
	Black	2.93	<0.01	2.64	3.25	3.17	<0.01	2.86	3.52
	Mixed and multiple	4.66	<0.01	3.85	5.64	4.82	<0.01	3.98	5.83
	Other	3.53	<0.01	3.13	3.98	3.62	<0.01	3.21	4.08
	Unknown	1.35	<0.01	1.25	1.47	1.35	<0.01	1.24	1.46
Sex	Male	1.00				1.00			
	Female	1.24	<0.01	1.19	1.28	1.24	<0.01	1.19	1.28
Socioeconomic status	1–most affluent	1.00				1.00			
	2	0.97	0.30	0.91	1.03	0.98	0.40	0.92	1.03
	3	1.06	0.06	1.00	1.12	1.10	<0.01	1.03	1.16
	4	1.17	<0.01	1.11	1.25	1.28	<0.01	1.21	1.35
	5–most deprived	1.27	<0.01	1.20	1.35	1.41	<0.01	1.33	1.50
Year of diagnosis		1.00	0.99	0.99	1.01	1.01	0.35	0.99	1.02

CRCcolorectal cancer

**Figure 2:**
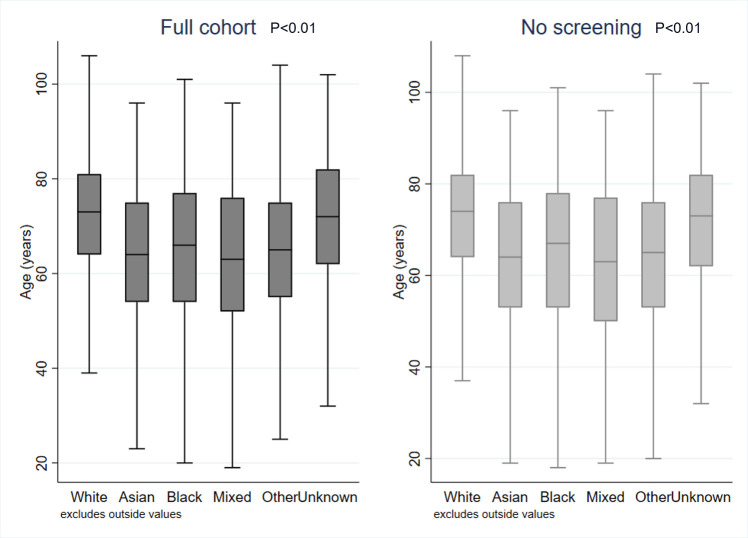
Median age at CRC diagnosis, by ethnic group (shown for the full cohort and excluding those diagnosed through screening). CRC, colorectal cancer.

The age profile of the CRC population varied by ethnic group, with a higher proportion of individuals from the white ethnic group being aged 60 and over than any other ethnic group (figure 1). The white ethnic group had the highest median age at onset (white: 73, IQR 64–81; Asian: 64, IQR 54–75; black: 66, IQR 54–77; mixed and multiple: 63, IQR 52–76; other ethnic groups: 65, IQR 55–75; p<0.01) (figure 2). The differences were not explained by differential uptake of screening between ethnic groups, when individuals who were diagnosed through screening were excluded the results were comparable and remained statistically significant (p<0.01) (figure 2).

A higher proportion of individuals in the Asian, black, mixed and multiple and other ethnic groups were under the CRC screening age (under 60 years) at diagnosis (36.8%, 36.6%, 41.6% and 34.7%, respectively) compared with 16.3% in the white ethnic group (table 1).

### Route to diagnosis

**Figure 3:**
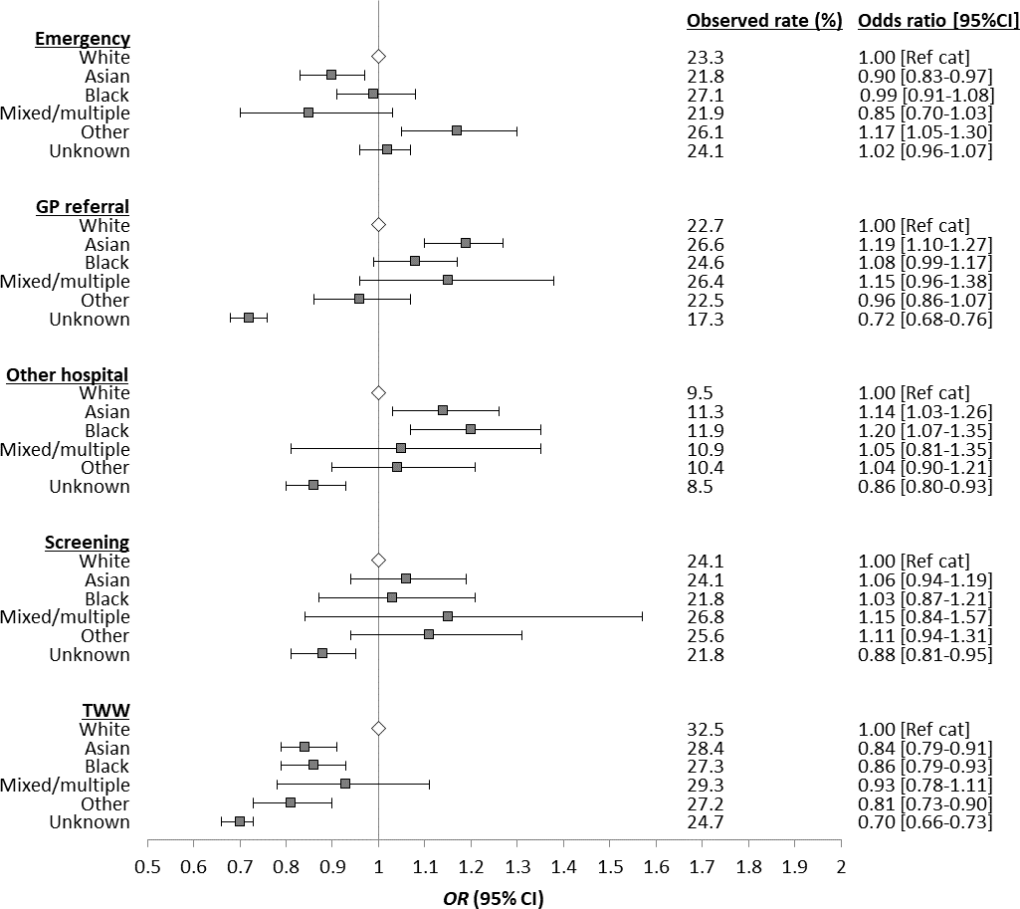
Results of multivariable models for the association between ethnic group and route to CRC diagnosis (each route modelled separately) (full model results available in online supplemental tables 1–5. Screening is restricted to individuals aged 60–74. CRC, colorectal cancer; GP, general practitioner.

Route to diagnosis varied by ethnic group, with individuals in the black ethnic group having the highest proportion of emergency diagnoses (27.1%) (figure 3). After adjustment for patient and tumour characteristics, this was not statistically significant when compared with the white ethnic group (OR 0.99, 95% CI 0.91 to 1.08) (figure 3). The Asian ethnic group had the lowest proportion of emergency diagnoses (21.8%) and this was statistically significant following adjustment when compared with the white ethnic group (OR 0.90, 95% CI 0.83 to 0.97) (figure 3). Diagnosis following a TWW referral was significantly less common among individuals from the Asian, black, other and unknown ethnic groups when compared with the white ethnic group (Asian OR 0.84, 95% CI 0.79 to 0.91, black OR 0.86, 95% CI 0.79 to 0.93, other OR 0.81, 95% CI 0.73 to 0.90 and unknown OR 0.70, 95% CI 0.66 to 0.73) (figure 3).

The highest proportion of diagnoses following a routine GP referral was observed in the Asian ethnic group (26.6%), and after adjustment, individuals from this group were 19% more likely to be diagnosed following a routine GP referral than their white counterparts (OR 1.19, 95% CI 1.10 to 1.27) (figure 3).

Among individuals aged 60 –74 at the time of CRC diagnosis, no statistically significant difference in diagnosis via screening was observed for any ethnic group when compared with the white ethnic group, with the exception of the unknown group (figure 3).

### Stage at diagnosis

The staging profile of the population varied by ethnic group, with metastatic disease (stage IV) present at diagnosis in over a quarter of individuals in the black, mixed and multiple and other ethnic groups (28.5%, 28.8% and 25.8%, respectively) compared with 22.9% in the white ethnic group (table 1). A higher proportion of early-stage CRC was observed in the white and Asian ethnic groups (39.9% and 38.8%, respectively) than in the black and unknown ethnic groups (34.9% and 33.4%, respectively) (p<0.01) (table 1).

**Figure 4:**
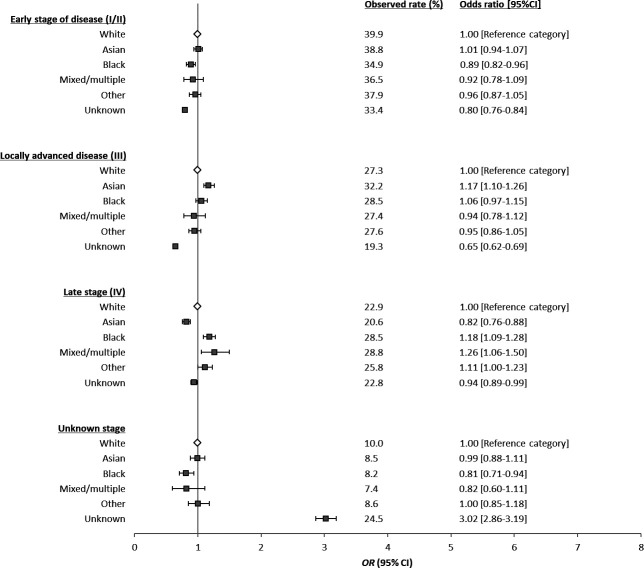
Results of multivariable models for the association between ethnic group and stage of CRC at diagnosis (full model results available in online supplemental tables 6–9). CRC, colorectal cancer.

Following adjustment for patient and tumour characteristics, it was demonstrated that individuals from both the black and unknown ethnic groups were significantly less likely than individuals from the white ethnic group to be diagnosed at an early stage (black OR 0.89, 95% CI 0.82 to 0.96, unknown OR 0.80, 95% CI 0.76 to 0.84). No significant difference remained for any other groups (figure 4). Locally advanced tumours (stage III) were significantly more common among individuals from the Asian ethnic group when compared with the white ethnic group (OR 1.17, 95% CI 1.10 to 1.26), the reverse was true for individuals from the unknown ethnic group (OR 0.65, 95% CI 0.62 to 0.69) (figure 4). Late-stage tumours (stage IV) were significantly more common among individuals from the black, mixed and multiple and other ethnic groups than the white ethnic group (black OR 1.18, 95% CI 1.09 to 1.28, mixed and multiple OR 1.26, 95% CI 1.06 to 1.50 and other OR 1.11, 95% CI 1.00 to 1.23) (figure 4). In contrast, they were significantly less common among individuals from the Asian ethnic group when compared with the white ethnic group (OR 0.82, 95% CI 0.76 to 0.88) (figure 4).

## Discussion

This large, population-level study within the English NHS identified significant differences in the timing and methods of CRC diagnosis across ethnic groups. While CRC is more common in the white ethnic groups, it demonstrated a younger age of onset in non-white ethnic groups than was observed in the white ethnic group. Differences in tumour site and stage of disease at diagnosis associated with ethnic group were also identified. It also showed significant differences in the route to CRC diagnosis, with individuals from non-white ethnic groups, particularly the Asian ethnic group, being more likely to be diagnosed via a routine GP referral and less likely to be diagnosed via TWW than their white counterparts.

The characteristics of individuals with CRC varied between ethnic groups, identifying the need to ensure that any targeted approach to outcome improvement considers this. The location of tumours within the colon and rectum differed between ethnic groups, with tumours of the rectum being most often seen in individuals from the Asian ethnic group and tumours of the right colon being seen most often in individuals from the black ethnic group, which reflects the findings of other studies.^25^ This difference in tumour site in relation to ethnic group may explain some of the differences in route to diagnosis observed, with right-sided tumours being associated with a higher risk of emergency diagnosis and tumours of the rectum associated with lower rates of emergency diagnosis.^26 27^ While there are known differences in tumour site related to sex,^28^ the differences in sex distribution seen in relation to ethnic group in this study are not large enough to account for the observed differences in tumour site. Tumours of the rectum and right colon are associated with different symptoms, presentations and stages at diagnosis. Studies have shown that rectal tumours often present after a long duration of symptoms in younger individuals.^29^

Advanced disease was present at diagnosis in a higher proportion of individuals from the black and mixed and multiple ethnic groups than was observed in the white and Asian ethnic groups, with late stage (stage IV) and locally advanced (stage III) disease accounting for 56.9% of all diagnoses in the black ethnic group. Other studies have examined this relationship but have used different ethnic groupings meaning that the results are not directly comparable; however, a study using data from England showed a significant increase in late-stage diagnosis of colon cancer among Caribbean men.^22^ Stage III disease was significantly more common in individuals from the Asian ethnic group than their counterparts in the white ethnic group while stage IV disease was significantly less common. This may be due to the higher prevalence of rectal and rectosigmoid cancers seen in the Asian ethnic group. Their behaviour in terms of the timing of onset of symptoms, the anatomic differences between the rectum and colon and their biology may have a bearing on these differences of staging at presentation. In addition, an increased likelihood of rectal bleeding as a mode of presentation for rectal tumours may be judged to overlap with benign anorectal bleeding, which may also account for some of the increased rate of routine GP referral in the Asian ethnic group, however, this requires further investigation.

The age profiles of the population with CRC differed between ethnic groups in this study with the white ethnic group being older than the non-white groups. Early-onset CRC carries similar challenges regardless of ethnic group; however, for non-white ethnic groups, it accounts for a larger proportion of CRC cases. These results identify a high prevalence of multiple characteristics associated with poor outcomes, namely early-onset CRC^30^ and increased levels of socioeconomic deprivation,^31^ all of which are a concern regardless of ethnicity but are more common in some of the non-white ethnic groups. Alongside recognising the differences in the characteristics of the background populations, it is also important to acknowledge that further investigation is needed to establish whether there are any significant differences in diagnostic risk factors^8 32 33^ or help-seeking behaviours and barriers^34^,^36^ between ethnic groups, which may explain some of the differences observed in this study.

Route to diagnosis was shown to be associated with ethnicity in this study, with individuals from all other ethnic groups being significantly less likely to be diagnosed through the TWW route, however, in contrast to other studies, no significant difference was identified for screening.^18^ Individuals from the Asian ethnic group were significantly more likely to be diagnosed following a routine referral from their GP than their white counterparts and individuals from the Asian and black ethnic groups were significantly more likely to be diagnosed via a routine inpatient or outpatient pathway than individuals from the white ethnic group. It is important to understand the reasoning behind this in order to identify any barriers to early diagnosis in this group. This may be particularly relevant with the introduction of quantitative faecal immunohistochemistry testing (FIT) guidelines^37^ and the introduction of a largely FIT-based urgent suspected CRC referral pathway. This study demonstrated an association between socioeconomic status and ethnic group, which requires further investigation. Further work is also required to determine whether the outcomes associated with each route to diagnosis are the same across ethnic groups and whether the association with socioeconomic status is consistent across groups.

The findings of this study demonstrate differences between ethnic groups in terms of the demographics of those diagnosed with CRC. The finding that those from non-white ethnic groups were more likely to be younger and live in more deprived areas may reflect the younger age and increased deprivation observed in these populations as a whole^38 39^ rather than an increased risk of cancer incidence in these populations. These characteristics are associated, with stage and route to diagnosis associated with ethnicity, route to diagnosis and socioeconomic status, it is important to acknowledge that these characteristics cluster and the relationships are difficult to disentangle. However, this demonstrates the importance of ensuring that any interventions seeking to improve outcomes in these populations address these demographic differences. This is a large, population-level study, including all individuals diagnosed with CRC within the English NHS. Due to the data used being retrospective, obtained from routinely collected healthcare data, the categorisation of ethnicity which was used has acknowledged issues.^2 40^ Broad ethnic categories (white, Asian, black, mixed and multiple, other and unknown) were used because the data were not powered to investigate subgroups (such as Indian, Pakistani or Bangladeshi), meaning that each group likely includes a heterogeneous population. This is a particular problem in the case of the other and mixed and multiple ethnic groups that are both heterogeneous and small, which is likely to affect the results. Further work should include subcategories and analysis of self-reported ethnicity.

The differences in the tumour and demographics observed between the ethnic groups in this study demonstrate the need for careful consideration when designing CRC services to ensure that both interventions and education meet the needs of the target population.

## supplementary material

10.1136/bmjgast-2024-001629 online supplemental file 1

## Data Availability

Data may be obtained from a third party and are not publicly available.
